# Cognitive Impairment in Long-Term Survivors of Testicular Cancer More Than 20 Years after Treatment

**DOI:** 10.3390/cancers13225675

**Published:** 2021-11-12

**Authors:** Johannes Stelwagen, Andrea T. Meuleman, Sjoukje Lubberts, Gerrie Steursma, Lara M. Kruyt, Jan W. Donkerbroek, Coby Meijer, Annemiek M. E. Walenkamp, Joop D. Lefrandt, Sandra E. Rakers, Rients B. Huitema, Marianne A. A. de Jong, Erwin M. Wiegman, Alfons C. M. van den Bergh, Igle J. de Jong, Joost A. Agelink van Rentergem, Sanne B. Schagen, Janine Nuver, Jourik A. Gietema

**Affiliations:** 1 Department of Medical Oncology, University Medical Center Groningen and University of Groningen, 9728 NT Groningen, The Netherlands; j.stelwagen@umcg.nl (J.S.); a.t.meuleman@umcg.nl (A.T.M.); s.lubberts@umcg.nl (S.L.); g.steursma@umcg.nl (G.S.); larakruyt@gmail.com (L.M.K.); j.donkerbroek1@amsterdamumc.nl (J.W.D.); j.meijer01@umcg.nl (C.M.); a.walenkamp@umcg.nl (A.M.E.W.); j.nuver@umcg.nl (J.N.); 2 Department of Internal Medicine, Division of Vascular Medicine, University Medical Center Groningen and University of Groningen, 9728 NT Groningen, The Netherlands; j.d.lefrandt@umcg.nl; 3 Department of Neuropsychology, University Medical Center Groningen and University of Groningen, 9728 NT Groningen, The Netherlands; s.e.rakers@umcg.nl (S.E.R.); r.b.huitema@umcg.nl (R.B.H.); 4 Department of Radiotherapy, Radiotherapeutic Institute Friesland, 8900 CC Leeuwarden, The Netherlands; m.a.a.jong@skf-rif.nl; 5 Department of Radiotherapy, Isala Hospital, 8025 AB Zwolle, The Netherlands; e.m.wiegman@isala.nl; 6 Department of Radiotherapy, University Medical Center Groningen and University of Groningen, 9728 NT Groningen, The Netherlands; a.c.m.van.den.bergh@umcg.nl; 7 Department of Urology, University Medical Center Groningen and University of Groningen, 9728 NT Groningen, The Netherlands; i.j.de.jong@umcg.nl; 8 Department of Psychosocial Research and Epidemiology, Netherlands Cancer Institute, 1018 WV Amsterdam, The Netherlands; j.agelink@nki.nl (J.A.A.v.R.); s.schagen@nki.nl (S.B.S.)

**Keywords:** testicular cancer, cancer survivors, late toxicity, neurocognitive impairment

## Abstract

**Simple Summary:**

Impaired cognition can be a late effect after treatment in long-term testicular cancer survivors, negatively affecting their daily life. However, little data is available beyond 20 years post-treatment. We assessed cognitive impairment in very-long-term survivors after treatment. In this study, we enrolled testicular cancer survivors with a follow-up duration ≥ 20 years—and age-matched healthy controls. Cognitive testing included the Auditory Verbal Learning Test, Letter Fluency Test, and Trail Making Test. We used fasting blood samples to assess the presence of hypogonadism and measured cardiovascular damage and aging parameters. We included 184 testicular cancer survivors (66 chemotherapy patients, 53 radiotherapy patients, and 65 orchiectomy only patients) and 70 healthy controls. The median follow-up was 26 years. Testicular cancer survivors performed worse on cognitive tests compared to controls. In univariate analysis, the presence of hypogonadism was associated with lower cognitive scores. Physicians and patients should be informed about timely cardiovascular risk management and testosterone supplementation therapy during follow-up to reduce the risk of cognitive impairment.

**Abstract:**

Background: Impaired cognition can be a late effect after treatment in long-term testicular cancer (TC) survivors, negatively affecting their daily life. However, little data is available beyond 20 years post-treatment. We assessed cognitive impairment in very long-term TC survivors after CT or RT and compared the results with stage I TC survivors and controls. Methods: In this cross-sectional multicenter cohort study, we enrolled TC survivors (treated with orchiectomy followed by CT or RT or orchiectomy only)—with a follow-up duration ≥ 20 years—and age-matched healthy controls. Cognitive testing included the Auditory Verbal Learning Test, Letter Fluency Test, Category Fluency Test, and Trail Making Test. We used fasting blood samples to assess the presence of hypogonadism and measured cardiovascular aging parameters, including carotid pulse wave velocity (c-PWV) and advanced glycation end products (AGEs). Results: We included 184 TC survivors (66 CT patients, 53 RT patients, and 65 orchiectomy-only patients) and 70 healthy controls. The median follow-up was 26 years (range: 20–42). TC survivors had a lower combined score of the cognitive tests (mean cumulative Z-score −0.85; 95% CI −1.39 to −0.33) compared to controls (mean 0.67; 95% CI −0.21 to 1.57, *p* < 0.01). In univariate analysis, the presence of hypogonadism (β −1.50, *p* < 0.01), high c-PWV (β −0.35, *p* = 0.09), and high AGEs (β −1.27, *p* = 0.02) were associated with lower cognitive scores, while only AGEs (β −1.17, *p* = 0.03) remained a significant predictor in multivariate analysis (Model R2 0.31, *p* < 0.01). Conclusions: Long-term TC survivors performed worse on cognitive tests compared to controls. Physicians and patients should be informed about timely cardiovascular risk management and testosterone supplementation therapy during follow-up to reduce the risk of cognitive impairment. Trial Registration: NCT02572934.

## 1. Introduction

Testicular cancer (TC) is the most common solid malignancy affecting males between the ages of 15 and 35 years [1]. Depending on the disease stage, TC is treated by either orchiectomy only or by orchiectomy followed by radiotherapy (RT) or platinum-based chemotherapy (CT). The TC prognosis is very good, even in cases of metastatic disease, with the 10-year survival rates reaching 80–90% [2,3]. This results in a still-growing number of TC survivors. However, adverse late treatment effects have been shown to result in increased morbidity from second cancers [4], cardiovascular disease (CVD) [5,6,7,8,9], nephrotoxicity [10,11], neurotoxicity [12], pulmonary toxicity [13], and Raynaud’s phenomenon [14,15]. Reports on cognitive impairment as a treatment effect, however, have shown a heterogeneous picture [16,17,18,19,20]. Several longitudinal studies reported an initial decline in cognitive function after treatment for TC with a return to baseline cognitive function after 12 months of follow-up [16,17]. Illustrating the diversity of the data, a cognitive assessment performed in TC survivors after a longer follow-up (2–7 years) reported 62.5% as cognitively impaired, exceeding the expected normative frequency of 25% [18]. Several other reports performed in long-term TC survivors with a median follow-up of 10–14 years confirm an increased long-term cognitive impairment in TC survivors treated with CT [19,20]. Therefore, it was hypothesized that the improvement in cognitive function after an initial decline following treatment is transient, and cognitive function may worsen again after longer follow-up [19].

The underlying mechanism of this worsening is still unclear but may be related to healthy tissue damage, such as vascular damage, induced directly by platinum-based CT or indirectly by increased CVD risk factors after CT [21,22]. TC survivors have a 1.6-fold higher risk of dying from CVD than the general population [23]. This vascular morbidity could be an important factor in the pathogenesis of increased cognitive impairment. In patients without a history of TC, vascular risk factors—such as higher fasting glucose and low-density lipoprotein (LDL) cholesterol—and advanced glycation end products (AGEs) are associated with a decline in cognitive function [24,25,26,27]. In TC survivors, however, such associations with cognitive impairment have not been extensively studied. Additionally, little information is available on objective cognitive performance of very long-term TC survivors. Therefore, in comparison to age-matched controls, we assessed the presence of cognitive impairment in a large group of long-term (>20 years) TC survivors treated with orchiectomy only, or with orchiectomy followed by radiotherapy or platinum-based chemotherapy. We aimed to objectify long-term cognitive impairment in this group and identify treatment-associated factors related to the development of these late effects. This is relevant to survivorship care and can support rehabilitation programs for long-term TC survivors.

## 2. Patients and Methods

### 2.1. Patients

We performed a multicenter, observational cross-sectional cohort study comprising three groups of TC survivors based on treatment modality and an age-matched control group. For the first TC group, we randomly selected 70 TC survivors from the institutional database who had previously been treated with both orchiectomy and CT (CT group) at the University Medical Center Groningen (UMCG). For the second group, 57 age-matched patients who had been treated with orchiectomy and RT (RT group) at either the UMCG, the Isala Hospital, or the Radiotherapeutic Institute Friesland (RIF) were included. For the third group, 70 age-matched patients who had been treated with orchiectomy only were included. The inclusion criteria for the TC survivor groups were: <40 years of age at diagnosis, <70 years of age at inclusion in the current study, and treatment for TC more than 20 years ago. The CT group included TC patients with good or intermediate prognosis according to the International Germ Cell Consensus Classification Group [28]. Patients receiving RT or CT for any other indication were not eligible. Participants with an active psychiatric disorder were excluded. The age-matched male controls were recruited with flyers distributed around the UMCG, especially in the non-patient areas and in neighboring supermarkets. Controls were <70 years of age. Exclusion criteria were an active episode of depression or previous treatment with CT, RT, or hormone therapy for any type of cancer. The study protocol was approved by the Ethics Committee at the University Medical Center Groningen (METc 2015/197). Written informed consent was obtained for all participants. The study was performed in accordance with the Declaration of Helsinki.

### 2.2. Assessments

Anthropometrics (weight, height, waist and hip circumference) and blood pressure were measured in a standardized manner. Fasting blood samples were used to assess the lipid profile (total cholesterol, triglycerides, high-density lipoprotein (HDL) and low-density lipoprotein (LDL)), glucose and HbA1C levels, high-sensitivity C-reactive protein (hs-CRP), creatinine levels, and the presence of either primary or secondary hypogonadism. Biologically active testosterone was calculated according to the validated method published by Vermeulen and colleagues [29].

To improve the homogeneity of study methods and enable between-study comparisons, cognitive function was assessed according to the recommendations from the International Cognition and Cancer Task Force (ICCTF) [30]. We therefore performed an Auditory Verbal Learning Test (AVLT)—including a delayed recall (DR)—[31], the Letter Fluency Test (LFT) [32], the Category Fluency Test (CFT), and the Trail Making Test (TMT) parts A and B [33]. A detailed description of these tests is provided in Supplementary Methods. All assessments were performed at the UMCG.

Cognitive impairment was defined according to the recommendations from the ICCTF—with either one test score below −2.0 standard deviations (SD) from the normative mean or two test scores below −1.5 SDs from the normative mean [30]—taking into account the expected probability of cognitive impairment due to the use of multiple cognitive tests [34]. Normative means were derived from the Advanced Neuropsychological Diagnostics Infrastructure (ANDI), an online normative database created from control datasets [35], which provides robust control data.

We assessed concurrent anxiety and depression complaints with the Hospital Anxiety and Depression Scale (HADS) questionnaire [36] and perception of cognitive dysfunction in daily life with the Cognitive Failure Questionnaire (CFQ) [37].

Vascular function and structure measurements were performed by specialized technicians at the vascular laboratory of the UMCG. Pulse wave velocity—an indicator of vascular stiffness—was measured over the carotid-femoral trajectory (cf-PWV) and locally at the carotid (c-PWV), while simultaneously measuring arm blood pressure (SphygmoCor). The carotid artery intima media thickness (c-IMT) was measured with the MYLABTMONE (Esaote; Genoa, Italy) ultrasound system. c-IMT was measured at the far wall of the carotid artery. Advanced glycation end products (AGEs) were estimated non-invasively using skin autofluorescence with the AGE reader [38]. Fasting blood samples were drawn to assess biochemical markers for vascular damage (von Willebrand factor), coagulation markers (i.e., FVIII, fibrinogen, plasminogen activator inhibitor-1 (PAI-1) antigen, and tissue plasminogen activator (tPA)) and hs-CRP.

### 2.3. Statistical Analysis

Z-scores—derived from ANDI—were normally distributed, so we reported means and standard deviations (SD). Differences in Z-scores between study groups were tested with a Student’s *t*-test for two groups or an ANOVA for comparison of more than two groups. When assessing presence of cognitive impairment, the probability of a false-positive result—due to multiple comparisons—was calculated according to the methodology published by Ingraham and Aiken [34]. Due to the use of six cognitive tests, the probability of scoring at least one test −2 SD from a normative mean is approximately 0.14. The probability of scoring at least two tests −1.5 SD from a normative mean is approximately 0.06; these frequencies should therefore be expected in any control group. Multivariate normative comparisons (MNC) were used to compare multiple cognitive tests simultaneously [39], with a cut-off *p*-value of 0.05 indicating cognitive impairment.

Multivariate regression analysis was performed on the cumulative Z-scores on cognitive function tests using a backwards stepwise method. Variables with a significant association on univariate analysis (*p* < 0.1) were included in the model. Variables included in univariate analysis were known or suspected cognitive denominators—such as age and anxiety or depressive symptoms—as well as cardiovascular parameters (cf-PWV, c-IMT, AGEs, c-PWV, vWF, FVIII, fibrinogen, PAI-1 antigen, and tPA), metabolic parameters (body mass index (BMI) and presence of metabolic syndrome) and endocrine parameters (testosterone levels and presence of hypogonadism). The data were analyzed with SPSS 23.0 (IBM-SPSS, Chicago, IL, USA).

## 3. Results

**Figure 1:**
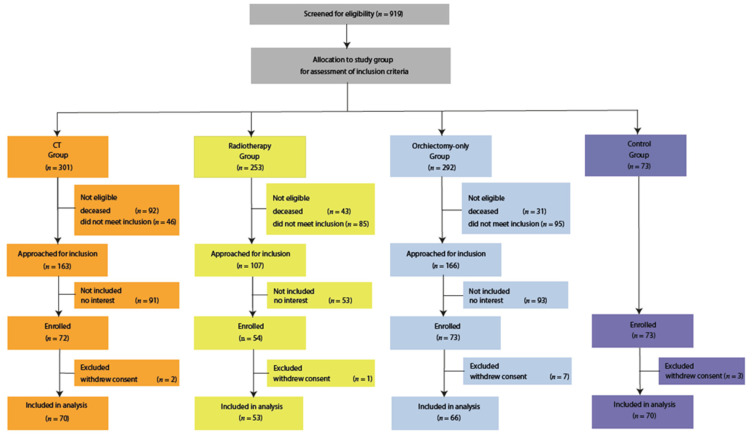
Consort diagram. The institutional database of testicular cancer patients treated at the University Medical Center of Groningen (UMCG) was used to identify testicular cancer survivors (TCS) treated with chemotherapy (CT). Patients were randomly selected and screened for inclusion. Eligible CT patients were approached if they were still alive and met the inclusion criteria: <40 years of age at diagnosis, <70 years of age at inclusion in the current study, treatment for TC was ≥20 years ago, and patients were treated with CT for either good or intermediate prognosis according to the International Germ Cell Consensus Classification (IGCCCG). This led to screening 301 CT patients, of which 138 were not eligible; 91 of the eligible patients declined to participate in the study. Next, the institutional databases of the UMCG, the Isala Hospital, and the Radiotherapy Institute Friesland were used to identify TCS treated with radiotherapy. Eligible RT patients were approached if they were still alive and met the inclusion criteria: <40 years of age at diagnosis, <70 years of age at inclusion in the current study, treatment for TC was ≥20 years ago. This led to screening 253 CT patients, of which 127 were not eligible; 53 of the eligible patients declined to participate in the study. This resulted in 23 included patients from the UMCG, 7 from the Isala Hospital, and 23 from the RIF. Lastly, the institutional database of the UMCG was used to identify TCS treated with orchiectomy only. Patients were age-matched to the CT group and approached for inclusion if they were alive and met the inclusion criteria: <40 years of age at diagnosis, <70 years of age at inclusion in the current study, treatment for TC was ≥20 years ago, and patients were not treated with CT or RT for any indication. This led to screening 292 orchiectomy-only patients, of which 126 were not eligible; 93 of the eligible patients declined to participate in the study. Furthermore, 70 age-matched healthy controls were included.

From August 2015 till February 2020, we included 66 TC survivors treated with orchiectomy and CT, 53 treated with orchiectomy and RT, 65 treated with orchiectomy only and 70 age-matched controls (Figure 1). Patient demographic, clinical, and laboratory characteristics at follow-up are reported in Table 1. There were no significant differences between TC survivors and the controls in age, level of education, current rate of employment, smoking behavior, blood pressure, total cholesterol, serum glucose, HbA1c levels and score of depressive symptoms on the HADS questionnaire. TC survivors had higher BMI compared to the controls (Table 1). Metabolic syndrome was more prevalent in TC survivors than in controls (OR 2.6 (95% CI, 1.3–5.1)). The total levels of testosterone were lower in TC survivors than in controls (median 12.8 nmol/L (range 2.9–35.8) vs. 15.5 nmol/L (range 7.2–53.8), *p* < 0.01). The RT group had a somewhat shorter duration of follow-up (Table 1). The CT group experienced more anxiety symptoms measured by the HADS questionnaire than the RT or orchiectomy only groups (median 4 (range 0–15) vs. 2 (range 0–14) vs. 3 (range 0–14), respectively, *p* = 0.03).

### 3.1. Cognitive Function—Individual Tests

**Figure 2:**
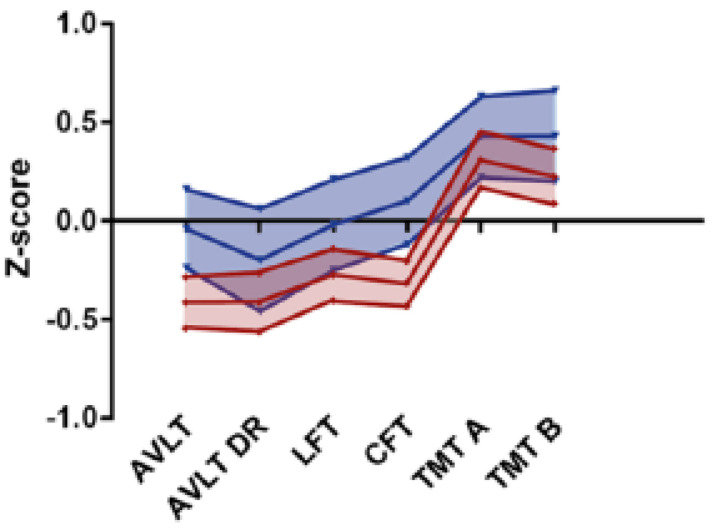
Median Z-scores and 95% confidence intervals—calculated from normative means derived from the Advanced Neuropsychological Diagnostics Infrastructure (ANDI)—for testicular cancer survivors (red) and healthy controls (blue). Abbreviations: AVLT, auditory verbal learning test; AVLT DR, auditory verbal learning test delayed recall; LFT, letter fluency test; CFT, category fluency test; TMT, trail making test. TC survivors scored worse than controls on the AVLT (mean Z-score −0.41 (95% CI: −0.54 to −0.28) vs. −0.04 (95% CI: −0.24 to 0.16), *p* < 0.01), the LF (mean Z-score −0.27 (95% CI: −0.40 to −0.14) vs. −0.02 (95% CI: −0.25 to 0.21), *p* = 0.04), and on both categories of the CFT (animals: mean Z-score −0.31 (95% CI: −0.43 to −0.19) vs. 0.03 (95% CI: −0.19 to 0.25), *p* < 0.01, profession: mean Z-score −0.33 (95% CI: −0.46 to −0.19) vs. 0.15 (95% CI: −0.14 to 0.43), *p* < 0.01).

TC survivors scored worse than controls on the AVLT, the LF, and on both categories of the CFT (Figure 2, Table 2). There were no differences in mean Z-scores between treatment groups of TC survivors (Table 2). With the exception of the AVLT DR, the CT group had the lowest scores on each test among TC survivors. Compared to the orchiectomy-only group, only the lower TMT-A scores in the CT group reached statistical significance in the post hoc analysis (mean Z-score 0.14 (95% CI: −0.07 to 0.37) vs. 0.51 (95% CI: 0.28 to 0.74), *p* = 0.03). All raw study scores are reported in Supplementary Table S1. Single test results more than 2 SD below average occurred as frequently in TC survivors—varying from 3% to 6% depending on the test—as in controls (Supplementary Table S2). Median scores on the CFQ were similar between TC survivors and controls (median 28 points (range 5–54) vs. median 28 points (5–62), *p* = 0.78). All CFQ subscores per study group are reported in Supplementary Table S3.

### 3.2. Cognitive Impairment—Cumulative Scores

**Figure 3:**
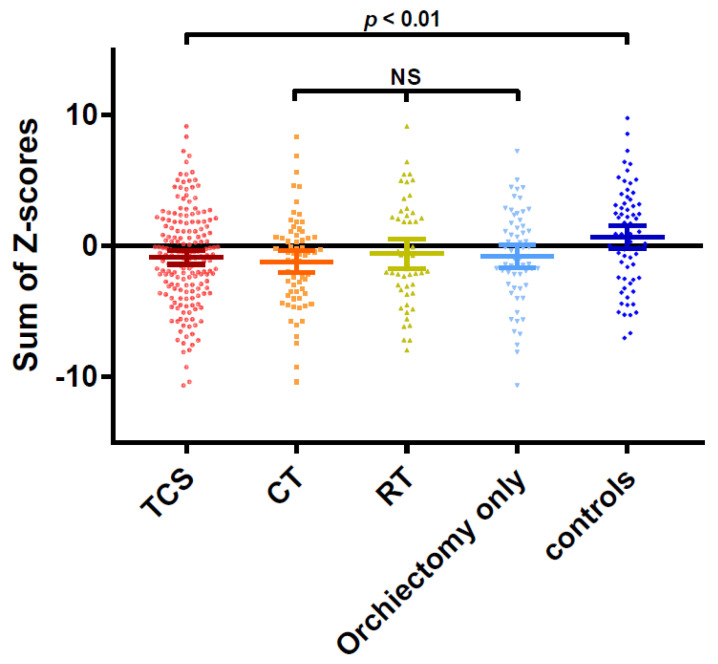
Sum of Z-scores on cognitive tests per study group. Each dot represents the sum of Z-scores on all cognitive tests for one individual. Bars represent median and 95% confidence intervals. Median sum of Z-scores was worse for TC survivors (mean −0.85; 95% CI −1.39 to −0.33) compared to the controls (mean 0.67; 95% CI −0.21 to 1.57, *p* < 0.01). No statistically significant differences were found between different treatment modalities for TC. Abbreviations: NS, not significant; TCS, testicular cancer survivors; CT, chemotherapy group; RT, radiotherapy group.

Regarding the mean sum of Z-scores (six different tests), TC survivors scored negative (mean −0.85; 95% CI −1.39 to −0.33) while controls scored positive (mean 0.67; 95% CI −0.21 to 1.57, *p* < 0.01). The sum of Z-scores was similar between the treatment modalities for TC (Figure 3). Subjective cognitive complaints reported through the CFQ did not correlate with the sum of Z-scores on the objective cognitive functioning tests (Spearman Rho −0.09, *p* = 0.18). Cognitive impairment was found in 16% of TC survivors and in 10% of controls (*p* = 0.24). The expected frequency of impairment in any control group was 17%. Using the multivariate normative comparisons—thus taking into account correlations between different cognitive tests—six participants were classified as cognitively impaired; five were TC survivors (3%), and one was from the control group (1%).

### 3.3. Confounders

Based on univariate regression, TC-associated factors—such as having received chemotherapy, the presence of hypogonadism, and lower biologically available testosterone levels—were associated with worse cumulative Z-scores on cognitive tests. RT was not associated with poorer performance on cognitive function. Age, symptoms of anxiety or depression, von Willebrand factor, AGEs and c-PWV were included in the multiple regression analysis (Table 3). In the multiple regression analysis on the sum of Z-scores of cognitive functioning tests including all participants, anxiety symptoms, a history of TC, levels of biologically available testosterone, and AGEs were independent factors (model R2 0.31, *p* < 0.01, Table 3). Other factors that were derived from the univariate analysis—such as age, depressive symptoms, von Willebrand factor, presence of primary hypogonadism, c-PWV, and having received chemotherapy for TC—did not significantly contribute to the model.

## 4. Discussion

In this study of long-term TC survivors, we focused on cognitive impairment after a median follow-up duration of 26 years by testing cognitive function and relating this to treatment modalities and potential causal factors, such as hypogonadism and cardiovascular risk factors. TC survivors—treated with either orchiectomy only or orchiectomy and radiotherapy or platinum-based chemotherapy—performed worse on cognitive tests compared to controls. In particular, they scored poorly on all language-oriented cognitive tests. This is in line with an epidemiological study of Swedish TC survivors treated with platinum-based chemotherapy (*N* = 960). It reported an increased incidence of long-term compromised language after a median follow-up of 11 years [40]. They also reported that CT-treated TC survivors were most affected. In our study, univariate analysis indicated that the administration of CT was the only predicting treatment modality for cognitive function. This effect disappeared in multivariate analysis, which was likely due to the small differences between all three treatment modalities.

Treatment with chemotherapy has been hypothesized to cause cognitive impairment—the so-called ‘chemobrain’—and has been widely studied in short- and long-term survivors of various types of cancer. During CT treatment for TC, an initial decline of cognitive function is seen, followed by normalization of cognitive performance after 6 to 18 months post-treatment [17,41]. Studies in long-term CT-treated TC survivors with median follow-up durations of 11 to 14 years also reported compromised cognitive function [20,42]. CT is associated with greater cardiovascular risks and could consequently cause cognitive impairment through vascular damage [43]. Platinum remnants are known to circulate in plasma for more than 10 years after cisplatin treatment, which could be a causal factor for long-term cognitive decline [44]. However, the results in our study did not show that very long-term CT-treated TC survivors performed significantly worse on a cognitive test compared to other treatment modalities. It might be that cognitive dysfunction is not that severe among very-long term CT-treated TC survivors (>20 years) compared to survivors of other treatment modalities.

TC survivors have an enhanced risk of developing early cardiovascular disease [45]. This “accelerated vascular aging” could be a factor in the decreased cognitive performance shown in our study. For example, c-PWV—a known marker for carotid stiffness—was associated with poorer cognitive function in univariate analysis. Previous studies showed a similar association between c-PWV and cognitive impairment [46,47,48], with data indicating that carotid stiffness is an early marker to identify a disturbance in the organization of the functional brain network, even before clinical vascular pathology occurs [48]. Moreover, the accumulation of AGEs—which are linked to cognitive impairment [25,26,27]—were also associated with poorer cognitive function in the current study and remained an independent predictor in multivariate analysis. This is in line with the “accelerated cognitive aging” reported in a prospective study of 920 community-dwelling elderly without life-threatening cancer diagnosis whose AGEs and cognitive function were periodically assessed for nine years. In these elderly, high levels of AGEs were associated with greater cognitive decline [27].

Since hypogonadism is associated with cognitive decline, several epidemiological studies have suggested an endocrine mechanism: the presence of low serum testosterone after treatment for TC [42,49]. Primary hypogonadism—either successfully compensated for by elevated LH (subclinical) or incompletely corrected—almost only occurred in our TC survivor cohort. In line with this, primary hypogonadism was associated with worse cognitive outcomes on univariate analysis. Although this association disappeared during multivariate analysis, biologically available testosterone levels remained associated with cognitive function. This is in line with our observation of cognitive decline across all treatment groups, since orchiectomy and primary hypogonadism was present to a certain extent in all TC survivors, irrespective of treatment modality. Thus, chronic hypogonadism might be an important underlying mechanism of cognitive impairment among very long-term TC survivors since both hypogonadism and cognitive impairment was seen in all three treatment modality groups. Although hypothesized that the CT-treated survivors would be at highest risk, this study does not support this hypothesis for very long-term TC survivors (>20 years post-treatment).

Testosterone supplementation to treat cognitive impairment is an intervention possibility but has shown mixed results in previous studies. A double-blind trial randomized 308 healthy men without a history of TC, aged 60 and older with low or low-to-normal testosterone concentrations, to receive either testosterone (*n* = 156) or placebo (*n* = 152). No improvement in cognitive function was found [50]. However, other placebo-controlled trials that included hypogonadal men without a history of TC with both low serum testosterone and cognitive impairment did report beneficial effects from testosterone replacement therapy compared to placebo [51,52]. Therefore, to consider testosterone supplementation, it seems important to only treat patients with cognitive impairment. Two randomized controlled trials to determine the effects of testosterone supplementation on complaints of hypogonadism are currently recruiting TC patients with low serum testosterone after treatment (NCT02991209 and NCT03339635). The results of these ongoing studies could clarify the potential benefits of testosterone supplementation on cognitive function in TC survivors.

Both these mechanisms—endocrine and vascular—might also explain why our CT-treated cohort performed slightly worse (although not reaching significance) on the cognitive tests than the orchiectomy-only and RT-treated TC survivors, since CT-treated survivors are more prone to various endocrine and vascular sequelae. However, the number of TC survivors with cognitive impairment—using the suggested definition from the ICCTF—did not exceed the statistically expected proportion of 17%. This could partly be due to the use of discrete cut-off values for cognitive impairment, which prevents more nuanced assessment of the continuous spectrum of cognitive function. Bottom-line, it is a reassuring finding that cognitive dysfunction among TC survivors is not highly present and thus may not be a big issue in very long-time survivors. However, there may be an underestimation of cognitive dysfunction by a limitation in our study that our results could be influenced by selection bias because we had no data on cognitive dysfunction from deceased patients. They might have died earlier from treatment-related causes during follow-up, such as vascular damage, and this might contribute to the underestimation of the presence and burden of cognitive dysfunction in TC survivors. Future prospective studies within this area are needed to address to what extent cognitive impairment is present in TC survivors over time.

## 5. Conclusions

Our study of a large cohort of TC survivors with uniquely long-term follow-up showed that TC survivors perform significantly worse than controls on a series of cognitive function tests. Although this effect size was clinically small, it was present in all treatment groups, especially the CT-treated patients. It is suggested that vascular and endocrine pathophysiological components underlie the long-term cognitive decline in TC survivors irrespective of their treatment. Timely cardiovascular risk management and testosterone supplementation therapy in selected TC survivors at high risk for late effects is relevant and may alleviate long-term cognitive decline. This needs to be further investigated.

## Figures and Tables

**Table 1:** Patient Demographic, Clinical, and Laboratory Characteristics at Follow-up According to Study Group.

	Testicular Cancer Survivors (TCS)									Healthy Controls		
Characteristic	All TCS *N* = 184		CT *N* = 66		RT *N* = 53		Orchiectomy Only *N* = 65		CT vs. RT vs. Orchiectomy Only	Controls *N* = 70		TCS vs. Controls
	*N*%		*N*%		*N*%		*N*%		*p*-Value	*N* %		*p*-Value
**Age, years**									0.24			0.76
					
**At study visit**												
**Median**	57		56		58		56			57		
**Range**	39–70		41–68		42–67		39–70			41–69		
**FU duration, years**									< 0.01	-		NA
**Median**	26		27		23		27					
**Range**	20–42		20–39		20–33		20–42					
**Clinical stage †**									< 0.01	-		NA
**I**	65	35	-	-	-	-	65	100		-		
**II**	87	47	38	58	49	92	-	-		-		
**III**	11	6	7	11	4	8	-	-		-		
**IV**	21	12	21	31	-	-	-	-		-		
**Level of education ¥**									0.82			0.72
**Median**	5		5		5		5			5		
**Range**	1–7		1–7		3–7		2–7			3–7		
**Employed**												
**Yes**	131	71	46	70	40	75	45	69	0.71	48	69	0.68
**Smoking behavior**									0.19			0.06
**Never smoked**	71	39	27	41	20	38	24	37		33	47	
**Former smoker**	57	31	24	36	16	30	17	26		27	39	
**Current smoker**	46	25	14	21	11	21	21	32		8	11	
**Unknown**	10	5	1	2	6	11	3	5		2	3	
**Blood pressure, mmHg**												
**Systolic**												
**Mean**	140		141		141		138		0.47	139		0.68
**SD**	15		14		17		14			15		
**Diastolic**												
**Mean**	88		90		88		86		0.16	87		0.55
**SD**	10		10		12		9			11		
**BMI, kg/m²**									0.44			0.03
**Median**	26.5		25.9		26.4		26.9			25.6		
**Range**	20.7–42.5		21.4–36.4		21.0–36.0		20.7–42.5			20.0–37.8		
**Total cholesterol**									0.46			0.57
**Mean**	5.2		5.2		5.2		5.0			5.2		
**SD**	1.2–9.2		3.2–7.7		3.2–6.9		1.2–9.2			3.4–7.8		
**Serum glucose, mmol/L**									0.10			0.33
**Median**	5.8		5.8		6.0		5.7			5.8		
**Range**	5.0–14		5.0–15.0		5.0–19.0		5.0–9.0			5.0–14.0		
**HbA1c, %**									0.10			0.06
**Median**	5.5		5.5		5.6		5.5			5.4		
**Range**	2.7–11.8		4.9–11.3		2.7–11.8		4.6–7.4			4.8–8.0		
**Metabolic syndrome #**	69	38	28	42	20	38	21	32	0.49	13	19	< 0.01
**Total testosterone, nmol/L**												
**Median**	12.8		11.3		12.9		14.1		0.11	15.5		< 0.01
**Range**	2.9–35.8		2.9–26.5		6.4–27.1		5.6–35.8			7.2–53.8		
**Bio-available testosterone, nmol/L**												
**Median**	5.9		5.6		6.1		6.3		0.08	6.9		< 0.01
**Range**	1.4–14.0		1.4–10.4		2.2–10.5		3.5–14.0			3.6–12.4		
**Serum LH, U/L**												
**Median**	7.9		10.3		7.9		7.1		0.01	4.6		< 0.01
**Range**	2.0–47.0		2.0–46.6		2.3–43.2		2.6–47.0			1.3–12.4		
**Testosterone therapy**	6	3	5	8	1	2	-	-	0.01	-	-	0.19
**Corrected prim. Hypogonadism ††**	42	23	18	27	13	25	11	17	0.11	4	6	< 0.01
**Uncorrected prim. Hypogonadism ¥¥**	33	18	17	26	8	15	8	12	0.35	0	0	< 0.01
**Secondary hypogonadism ##**	33	18	12	18	9	17	12	18	0.89	11	16	0.67
**Depressive symptoms (HADS)**									0.47			0.20
**Median**	3		3		2		2			2		
**Range**	0–12		0–12		0–9		0–12			0–14		
**Anxious symptoms (HADS)**									0.03			0.86
**Median**	3		4		2		3			3		
**Range**	0–15		0–15		0–14		0–14			0–13		

Abbreviations: TCS, testicular cancer survivors; CT, chemotherapy group; RT, radiotherapy group; HbA1c, glycated hemoglobin; LH, luteinizing hormone; HADS, Hospital Anxiety and Depression Scale; FU, follow-up; SD, standard deviation; BMI, body mass index; † According to Royal Marsden classification; ¥ Classification by Verhage, 1964; # Definition according to NCEP ATP III criteria; †† corrected, subclinical, primary hypogonadism was defined as testosterone >10.9 nmol/L and LH > 8.6 IU/L; ¥¥ uncorrected primary hypogonadism was defined as serum testosterone <10.9 nmol/L and LH> 8.6 IU/L or treatment with testosterone supplementation; ## secondary hypogonadism was defined as serum testosterone <10.9 nmol/L and LH <8.6 IU/L.

**Table 2:** Mean Z-scores on Cognitive Tests per Group.

	Testicular Cancer Survivors (TCS)					Healthy Controls	
Characteristic	All TCS *N* = 185	CT *N* = 66	RT *N* = 53	Orchiectomy Only *N* = 65	CT vs. RT vs. Orchiectomy Only *p*-Value †	Controls *N* = 70	TCS vs. Controls *p*-Value †
**AVLT**					0.78		< 0.01
**mean**	−0.41	−0.44	−0.35	−0.41		−0.04	
**95% CI**	−0.54 to −0.28	−0.66 to −0.22	−0.63 to −0.07	−0.62 to −0.21		−0.24 to 0.16	
**AVLT DR**					0.11		0.16
**mean**	−0.41	−0.29	−0.29	−0.62		−0.20	
**95% CI**	−0.56 to −0.26	−0.52 to −0.05	−0.57 to −0.01	−0.89 to −0.35		−0.46 to 0.06	
**LFT**					0.72		0.045
**mean**	−0.27	−0.32	−0.29	−0.20		−0.02	
**95% CI**	−0.40 to −0.14	−0.56 to −0.09	−0.56 to −0.02	−0.40 to −0.01		−0.25 to 0.21	
**CFT**					0.73		< 0.01
**mean**	−0.31	−0.34	−0.24	−0.34		0.10	
**95% CI**	−0.43 to −0.20	−0.54 to −0.14	−0.43 to −0.05	−0.54 to −0.14		−0.12 to 0.32	
**TMT-A**					0.11		0.36
**mean**	0.31	0.14	0.27	0.51		0.43	
**95% CI**	0.17 to 0.45	−0.07 to 0.37	−0.02 to 0.57	0.28 to 0.74		0.22 to 0.63	
**TMT-B**					0.26		0.12
**mean**	0.23	0.07	0.34	0.30		0.43	
**95% CI**	0.09 to 0.37	−0.15 to 0.29	0.07 to 0.62	0.05 to 0.55		0.20 to 0.66	

Abbreviations: TCS, testicular cancer survivors; CT, chemotherapy group; RT, radiotherapy group; AVLT, auditory verbal learning test; AVLT DR, auditory verbal learning test delayed recall; LFT, letter fluency test; CFT, category fluency test; TMT, trail making test; † Comparison of three groups was made with an ANOVA, comparison of two groups with students’ T test.

**Table 3:** Univariate and Multivariate Analysis on the Sum of Z-scores.

	Univariate Analysis			Multivariate Analysis			
	β	SE	*p*-Value	β	SE	*p*-Value	Model R
						**<0.01**	**0.31**
Constant				**7.47**	**2.20**	**<0.01**	
Age	**−0.06**	**0.032**	**0.06**	−0.04	0.04	0.38	
Smoking (pack years)	0.01	0.02	0.44				
Anxiety (HADS)	**−0.19**	**0.07**	**<0.01**	**−0.15**	**0.08**	**0.05**	
Depression (HADS)	**−0.19**	**0.08**	**0.02**	−0.03	0.13	0.83	
CFQ	−0.02	0.02	0.37				
HbA1C (%)	−0.41	0.32	0.21				
LDL	0.42	0.25	0.11				
Metabolic Syndrome (Y/N)	−0.48	0.50	0.35				
Hypertension (Y/N)	−0.76	0.66	0.25				
Fibrinogen	−0.11	0.38	0.78				
Von Willebrand Factor	**−0.01**	**0.006**	**0.08**	−0.003	0.006	0.63	
Factor VIII	−0.004	0.004	0.39				
t-PA antigen	0.03	0.04	0.34				
PAI antigen	0.01	0.01	0.18				
Testosterone (bio-available)	**0.32**	**0.12**	**<0.01**	**0.20**	**0.12**	**0.09**	
Primary Hypogonadism (Y/N)	**−1.50**	**0.50**	**<0.01**	−0.59	0.56	0.29	
hs-CRP	−0.02	0.06	0.73				
BMI	0.02	0.07	0.83				
Carotid-Femoral PWV	−0.10	0.15	0.50				
Advanced Glycation End Product	**−1.27**	**0.53**	**0.02**	**−1.17**	**0.54**	**0.03**	
Carotid Intima Media Thickness	−0.29	1.83	0.88				
Carotid PWV	**−0.35**	**0.19**	**0.09**	−0.29	0.21	0.16	
TC survivor (Y/N)	**−1.54**	**0.52**	**<0.01**	**−1.17**	**0.53**	**0.03**	
Chemotherapy (Y/N)	**−1.00**	**0.53**	**0.06**	−0.34	0.63	0.59	
Radiotherapy (Y/N)	−0.15	0.58	0.80				

## Data Availability

Data available on request due to restrictions eg privacy or ethical.

## Supplementary Materials

The following are available online at https://www.mdpi.com/article/10.3390/cancers13225675/s1: Supplementary Methods and Supplementary Tables available online. Table S1: Raw scores on cognitive tests per group., Table S2: Proportion impaired scores on cognitive tests per group., Table S3: All subscales of the Cognitive Failure Questionnaire per study group.
