# Supplementary material for: Cognitive Impairment in Long-Term Survivors of Testicular Cancer More Than 20 Years after Treatment
Source: Cancers (Basel). 2021 Nov 12;13(22):5675. doi: 10.3390/cancers13225675 (PMC8616311; doi:10.3390/cancers13225675)
Supplement: Supplementary file 1 [file cancers-13-05675-s001.zip › cancers-1464042-supplementary.pdf]

Cognitive Impairment in Long-Term Survivors of Testicular Cancer More Than 20 Years after Treatment

Johannes Stelwagen, Andrea T. Meuleman, Sjoukje Lubberts, Gerrie Steursma, Lara M. Kruyt, Jan Willem Donkerbroek, Coby Meijer, Annemiek M. E. Walenkamp, Joop D. Lefrandt, Sandra E. Rakers, Rients B. Huitema, Marianne A. A. de Jong, Erwin M. Wiegman, Alfons C. M. van den Bergh, Igle J. de Jong, Joost A. Agelink van Rentergem, Sanne B. Schagen, Janine Nuver and Jourik A. Gietema

1. Supplementary methods

1.1. Cognitive tests

The AVLT was used as an alternative for the Hopkins Verbal Learning Test —Revised (HVLT-R). The Dutch AVLT is based on the Rey AVLT (Rey, 1964) and measures learning and memory capabilities. Five presentations of a 15-word list are given, each followed by an attempted direct recall. Approximately 20 minutes after the direct recall an attempted delayed recall of the 15-word list takes place. Lastly, during the recognition test, the participant is presented with 30 words and asked to recognize which words were part of the 15-word list and which were not.[28] The fluency tests (LFT and CFT) measure aspects of language and executive function. The LFT is a validated Dutch version of the Controlled Word Association Test and uses three letters.[29] Individuals are given one minute to name as many words as possible beginning with one of the letters. This procedure is then repeated for the remaining two letters. During the CFT, participants have to produce as many words as possible from a category—animals (CFT-A) and professions (CFT-P)—during one minute. The TMT measures information processing speed and aspects of executive function.[30] It consists of two parts in which the subject is instructed to connect a set of 25 dots as quickly as possible. Cognitive test performance of our study participants were compared to the normative data from the normative database of Advanced Neuropsychological Diagnostics Infrastructure (ANDI). The number of individual control measurements included in the ANDI is large, varying from 1855 to 7583 per cognitive test. This provides robust control data for our analyses.

Table S1. Raw Scores on Cognitive Tests per Group.

Characteristic	Testicular Cancer Survivors (TCS)					Healthy Controls	
	**All TCS** *N* = 185	**CT** *N* = 66	**RT** *N* = 53	**Orchiectomy Only** *N* = 65	**CT vs. RT vs. Orchiectomy Only** *P*-value†	**controls** *N* = 70	**TCS vs. Controls** *P*-value†
RAVLT median range	38 11–60	39 22–60	37 11–59	39 24–55	0.83	42 24–64	< 0.01
RAVLT DR median range	7 0–14	8 2–14	7 2–14	7 0–13	0.31	9 0–14	0.08
LFT median range	33 15–65	31 15–59	33 15–60	36 15–65	0.32	36 17–78	0.09
CFT median range	40 17–67	39 17–67	40 23–61	40 19–63	0.68	42 25–83	< 0.01
TMT-A median	30 13–122	33 16–95	30 13–122	27 13–75	0.04	30 15–76	0.46

range							
TMT-B median range	68 27–294	72 36–294	64 32–219	63 27–172	0.26	67 24–184	0.22

Abbreviations: TCS, testicular cancer survivors; CT, chemotherapy group; RT, radiotherapy group; AVLT, auditory verbal learning test; AVLT DR, auditory verbal learning test delayed recall; LFT, letter fluency test; CFT, category fluency test; TMT, trail making test; † Comparison of three groups was made with an ANOVA if normally distributed, or Kruskal Wallis if not. Comparison of two groups with students' T test or Mann-Whitney U Test.

Table S2. Proportion Impaired Scores on Cognitive Tests per Group.

Characteristic	Testicular Cancer Survivors (TCS)					Healthy Controls	
	All TCS *N* = 185	CT *N* = 66	RT *N* = 53	Orchiec-tomy Only *N* = 65	CT vs. RT vs. Orchiec-tomy Only	controls *N* = 70	TCS vs. Controls
					P-value†		*P*-value†
AVLT proportion impaired (%)	2	2	4	2	0.64	0	0.58
AVLT DR proportion impaired (%)	6	3	2	11	0.06	7	0.59
LFT proportion impaired (%)	2	3	2	2	0.83	0	0.58
CFT proportion impaired (%)	3	3	0	5	0.30	0	0.16
TMT-A proportion impaired (%)	2	3	4	0	0.32	1	1.00
TMT-B proportion impaired (%)	2	3	2	0	0.39	1	1.00

Abbreviations: TCS, testicular cancer survivors; CT, chemotherapy group; RT, radiotherapy group; AVLT, auditory verbal learning test; AVLT DR, auditory verbal learning test delayed recall; LFT, letter fluency test; CFT, category fluency test; TMT, trail making test; † Proportions were compared using χ2-test.

Table S3. All Subscales of the Cognitive Failure Questionnaire per Study Group.

Characteristic	Testicular cancer survivors (TCS)					Healthy controls	
	All TCS N = 185	CT N = 66	RT N = 53	Orchiec-tomy only N = 65	CT vs. RT vs. Orchiec-tomy only	controls N = 70	TCS vs. controls
					P-value†		P-value†
**CFQ total score** median	24 0 —65	29 2 —49	22 0 —53	25 0 —65	0.12	27 0 —53	0.87

range							
CFQ —memory median range	6<br>0 —20	7<br>0 —17	5<br>0 —16	7<br>0 —20	0.12	6<br>0 —18	0.76
CFQ —distractibility median range	10<br>0 —24	10<br>0 —22	8<br>0 —21	10<br>0 —24	0.36	10<br>0 —18	0.86
CFQ —social blunders median range	6<br>0 —19	7<br>0 —14	6<br>0 —13	7<br>0 —19	0.39	6<br>0 —14	0.23
CFQ —forgetting names median range	4<br>0 —8	4<br>0 —7	3<br>0 —7	4<br>0 —8	0.26	4<br>0 —8	31

Abbreviations: TCS, testicular cancer survivors; CT, chemotherapy group; RT, radiotherapy group; CFQ, cognitive functioning index; † Comparison of three groups was made with an ANOVA if normally distributed, or Kruskal Wallis if not. Comparison of two groups with students' T test or Mann-Whitney U Test.
